# Adipokines and Vascular Modulators in CKM: Emerging Biomarkers as Diagnostic and Therapeutic Targets

**DOI:** 10.3390/ijms262211083

**Published:** 2025-11-16

**Authors:** Cezara-Andreea Gerdanovics, Șoimița-Mihaela Suciu, Olga-Hilda Orășan, Ioana Para, Vladiana-Romina Turi, Mircea-Vasile Milaciu, Mirela-Georgiana Perne, Lorena Ciumărnean, Nicoleta-Valentina Leach, Teodora-Gabriela Alexescu, Vlad-Dumitru Brata, Alexandru Gerdanovics, Angela Cozma

**Affiliations:** 1 Department of Internal Medicine, 4th Medical Discipline, “Iuliu Hațieganu” University of Medicine and Pharmacy, Republicii Street, No. 18, 400015 Cluj-Napoca, Romania; andreea.ceza.irimie@elearn.umfcluj.ro (C.-A.G.); hilda.orasan@umfcluj.ro (O.-H.O.); ioana.para@umfcluj.ro (I.P.); vasile.milaciu@umfcluj.ro (M.-V.M.); albmirela@yahoo.ro (M.-G.P.); teodora.alexescu@umfcluj.ro (T.-G.A.); angelacozma@yahoo.com (A.C.); 2 Department of Physiology, “Iuliu Hațieganu” University of Medicine and Pharmacy, 1-3 Clinicilor Street, 400006 Cluj-Napoca, Romania; 3 University Clinic of Internal Medicine IV, Faculty of Medicine, “Victor Babes” University of Medicine and Pharmacy of Timisoara, 2 Eftimie Murgu Square, 300041 Timisoara, Romania; turi.vladiana@umft.ro; 4 Department 2, Faculty of Nursing and Health Sciences, “Iuliu Hațieganu” University of Medicine and Pharmacy, Republicii Street, No. 18, 400015 Cluj-Napoca, Romania; lorena_ciumarnean@yahoo.com (L.C.); nicoleta_leach@yahoo.com (N.-V.L.); 5 Department of Gastroenterology, Regional Institute of Gastroenterology and Hepatology “Prof. Dr. Octavian Fodor”, 400394 Cluj-Napoca, Romania; brata_vlad@yahoo.com; 6 Department of Pathophysiology, “Iuliu Haţieganu” University of Medicine and Pharmacy, Victor Babeş Street, No. 2-4, 400012 Cluj-Napoca, Romania; alexandru.gerdanovics@elearn.umfcluj.ro; 7 Clinical Rehabilitation Hospital, Viilor Street, No. 46-50, 400066 Cluj-Napoca, Romania

**Keywords:** cardio-kidney-metabolic syndrome, adipokines, retinol-binding protein 4, lipocalin 2, apolipoprotein M, Klotho, matrix Gla protein, biomarkers, vascular calcification, oxidative stress

## Abstract

Cardio-kidney-metabolic (CKM) syndrome represents an integrated clinical and molecular continuum encompassing metabolic dysfunction, cardiovascular disease and chronic kidney disease. This multidimensional disorder arises from interdependent biological pathways that extend beyond conventional risk factors. Emerging evidence highlights a group of adipokines and vascular modulators—including retinol-binding protein 4 (RBP4), lipocalin 2 (LCN2), apolipoprotein M (ApoM), Klotho and matrix Gla protein (MGP)—emerging molecular modulators with potential involvement in CKM pathophysiology. Pro-inflammatory adipokines such as RBP4 and LCN2 contribute to insulin resistance, oxidative stress and endothelial dysfunction. In contrast, protective molecules including ApoM and Klotho preserve nitric oxide bioavailability, lipid metabolism and antioxidant defense. MGP modulates vascular calcification and adipose remodeling, with its inactive form (dp-ucMGP) linked to vascular stiffness and renal decline. The combined dysregulation of these molecules sustains cycles of inflammation, oxidative stress and tissue remodeling that drive CKM progression. Collectively, current data support their dual role as biomarkers and therapeutic targets. Nonetheless, clinical translation remains limited, emphasizing the need for standardized assays, longitudinal validation, and integrative multimarker approaches within precision medicine frameworks for CKM syndrome.

## 1. Introduction

Until recently, metabolic syndrome (MetS), cardiovascular disease (CVD) and chronic kidney disease (CKD), were often considered as distinct clinical entities, each of them being managed within its own specialty. However, growing evidence has highlighted the interdependence between these systems, leading to a more integrated understanding of their pathophysiology [1].

“Cardiorenal syndrome” is a concept used to describe the bidirectional relationship between heart and kidney dysfunction [2], marking the pathophysiological connection of acute and chronic heart and kidney diseases [3]. On the other hand, the connection between metabolic syndrome and kidney disease has long been recognized, with this synergistic relationship between the three creating a pathophysiological loop representing a growing concern for healthcare [4,5,6,7,8].

In the United States, more than one in four adults is affected by at least one component of the CKM triad, and globally, the prevalence of coexisting CKM-related conditions is estimated at 25–30%, highlighting the growing burden of this multimorbidity [9].

To a better understanding of how CKM syndrome develops and progresses, it is essential to explore the role of specific signaling molecules that mediate interactions between adipose tissue, cardiovascular system and kidneys. Recent research has highlighted a group of adipokines [10,11,12,13] and vascular modulators associated with cardio-kidney-metabolic regulation—including Klotho [14] and Matrix-Gla Protein MGP [15,16]—that are thought to influence this pathological process.

Adipose tissue is distributed throughout the body, predominantly in visceral and subcutaneous regions and plays a central role in maintaining health by synthesising and storing triglycerides within lipid droplets (lipogenesis), as well as releasing fatty acids into the bloodstream (lipolysis) during times of energy deficit. Beyond serving as an energy reservoir, adipose tissue secretes a variety of bioactive molecules that function through paracrine, autocrine, endocrine and vasocrine signaling to regulate metabolic balance. These molecules include a group of immunomodulatory proteins commonly known as ‘adipokines’ or ‘adipocytokines’ [10]. Alterations in adipokine expression are believed to contribute to the chronic low-grade inflammation observed in obesity. Both clinical and experimental research have documented the influence of adipokines on the inflammatory response, implicating them in the development of obesity-related conditions such as CVD [17]. Variations in metabolic function across different adipose depots result in different adipokine secretion profiles among individuals [18]. Obesity generally shifts this balance toward increased production of pro-inflammatory adipokines, regardless of depot location [9]. Pro-inflammatory adipokines —retinol-binding protein 4 (RBP4) and lipocalin 2 (LCN2)—typically predominate over anti-inflammatory mediators like apolipoprotein M (apoM). This pathogenic adipokine profile has been linked to the progression of CKM, renal disfunction and CVD associated with obesity [17,19].

Another adipokine-related protein associated with CKM regulation is Klotho, a key protein involved in mammalian aging, existing in multiple isoforms that each perform distinct functions [14,20]. The circulating soluble and secreted forms—collectively known as α-Klotho—are believed to exert endocrine and paracrine effects on several organs, including the kidneys, bones, brain, cardiovascular system, lungs and vascular endothelium [21,22,23,24]. Decreased levels of Klotho are commonly associated with aging [14,20]. Recent retrospective studies suggested that Klotho may serve as a promising biomarker for a range of age-related diseases such as heart failure, hypertension, diabetes and cardiovascular mortality [14]. Its involvement in aging and metabolic processes, along with its endocrine and paracrine actions on key organs such as the kidneys and cardiovascular system, suggests that Klotho may contribute to the link between adipose tissue dysfunction and CKM progression.

Other vascular regulators like MGP play an essential role in the CKM axis by inhibiting vascular calcification and maintaining arterial health [15]. Dysregulation of MGP, often influenced by metabolic and inflammatory states, contributes to the vascular complications commonly observed in CKM syndrome progression.

The selection of the five molecules (RBP4, LCN2/NGAL, ApoM, Klotho and Matrix Gla protein) was not arbitrary but guided by the CKM pathophysiology proposed in the 2023 AHA Scientific Statement [1], which highlights oxidative stress, inflammation, endothelial dysfunction and insulin resistance as core mechanisms. While classical adipokines (e.g., adiponectin, leptin, chemerin) primarily reflect adipose metabolic regulation and have been extensively reviewed, these five molecules represent emerging candidates increasingly reported across multiple organ systems.

In addition, these molecules represent the focus of an ongoing prospective research program conducted by our group, in which their diagnostic and prognostic potential will be investigated in patient cohorts. Thus, their inclusion in this review reflects both published emerging evidence and their relevance for hypothesis-driven future studies, rather than arbitrary selection. Throughout this manuscript we apply the 2023 American Heart Association nomenclature and use the term CKM syndrome. CKD, CVD and metabolic dysfunction are used only when referring to individual organ-specific conditions.

This group of adipokines and related regulatory proteins form a complex molecular system that links adipose tissue dysfunction to systemic metabolic, vascular and renal alterations. Understanding the sequential progression of the CKM syndrome is essential for identifying early biomarkers that can anticipate the transition between stages. As metabolic, cardiac and renal dysfunctions follow a progressive evolution, highlighting the underlying pathophysiological cascade can provide a focused insight into disease trajectory. This integrated view lays the foundation for hypothesis-generating research on biological risk stratification and supports exploration of molecular pathways for future early-intervention strategies.

## 2. Methods/Scope of the Narrative Review

This review is designed as a narrative synthesis, not as a systematic review. We did not perform protocol registration, risk-of-bias assessment, statistical pooling of effect estimates or quantitative meta-analysis. The purpose of this article is to integrate mechanistic and translational evidence and to generate hypotheses regarding the role of emerging biomarkers in CKM syndrome.

We conducted a focused narrative search in PubMed and Google Scholar between January 2015 and August 2025. 

Search terms included combinations of: “cardio-kidney-metabolic syndrome”, “RBP4”, “LCN2/NGAL”, “ApoM”, “Klotho”, “Matrix Gla protein”, together with “oxidative stress”, “vascular injury”, “endothelial dysfunction”, “renal damage”. 

We included: human studies (observational cohorts, prospective studies, meta-analyses, Mendelian randomization, when available), experimental studies on animal models or cell systems, when they contributed to understanding molecular pathways relevant to CKM and mechanistic studies exploring pathways linking these molecules to CKM.

We excluded case reports, non-original articles and studies lacking mechanistic or assay-related information. Screening was performed independently by two authors—disagreements were resolved by consensus. The purpose was not to perform a systematic review, but to synthesize mechanistic convergence and emerging translational evidence of these molecules within CKM.

As a narrative review, the methodology is subject to several inherent limitations, including potential selection bias and the lack of predefined quantitative eligibility criteria. Study heterogeneity prevented the application of systematic synthesis tools; therefore, conclusions should be interpreted as hypothesis-generating rather than definitive. The aim was to provide an integrated mechanistic overview, not to exhaustively evaluate all available evidence.

## 3. Integrated Pathophysiological Mechanisms in CKM Syndrome

The progression of CKM syndrome is driven by a complex interplay of interdependent molecular pathways. These molecules intersect at shared biological pathways, which could improve understanding of disease mechanisms and generate hypotheses for future therapeutic studies—Also, understanding these interconnected mechanisms is essential for identifying new therapeutic targets and refining risk stratification in CKM.

### 3.1. Oxidative Stress and Inflammation Leading to Endothelial Dysfunction and Vascular Calcification: Synergistic Effects of Protective and Harmful Pathways

Oxidative stress has long been recognized as a key contributor to the development of endothelial dysfunction. Experimental studies suggest that RBP4 activates the TLR4–NLRP3–NFκB inflammatory cascade, promoting ROS generation, mitochondrial dysfunction and reduced nitric oxide (NO) synthesis, ultimately triggering endothelial injury [25].

LCN2 further amplifies this redox–inflammatory axis by stabilizing MMP-9 and promoting extracellular matrix remodeling. Mechanistically, LCN2 impairs endothelial function by promoting eNOS uncoupling, reducing nitric oxide (NO) bioavailability and increasing oxidative stress. In murine models, LCN2 deficiency protects against high-fat diet-induced endothelial dysfunction, preserves eNOS dimerization, and maintains NO-mediated vasodilation. Conversely, administration of LCN2 induces endothelial dysfunction, characterized by impaired endothelium-dependent relaxation and enhanced endothelium-dependent contraction, partly via upregulation of cyclooxygenase (COX) and modulation of cytochrome P450-2C9 activity, which further increases reactive oxygen species production and vascular inflammation [26,27,28,29,30,31].

ApoM modulates endothelial dysfunction primarily through its role as a carrier of S1P on high-density lipoprotein (HDL) particles. ApoM/S1P complexes also exert anti-inflammatory effects by downregulating endothelial adhesion molecules such as VCAM-1 and E-selectin, thereby reducing leukocyte adhesion and transmigration during inflammation. This mechanism is more efficient when S1P is carried by ApoM-HDL compared to albumin, highlighting the specificity of the ApoM-S1P axis in vascular protection [32,33]. The ApoM-S1P axis is thus central to the maintenance of endothelial homeostasis, and its disruption contributes to the pathophysiology of endothelial dysfunction and atherosclerosis.

MGP plays a central role in preventing vascular calcification and maintaining endothelial function. The active, fully carboxylated and phosphorylated form of MGP inhibits vascular calcification through two principal mechanisms: direct binding to calcium-phosphate crystals, thereby preventing their deposition in the vascular wall, and inhibition of bone morphogenetic proteins (BMP-2 and BMP-4), which are key drivers of osteogenic differentiation in vascular cells. When MGP is undercarboxylated or dephosphorylated—typically due to vitamin K deficiency or antagonism—its inhibitory function is lost, leading to increased BMP signaling, upregulation of osteogenic transcription factors such as RUNX2 and transdifferentiation of vascular smooth muscle cells and endothelial cells into osteoblast-like cells, which accelerates calcification [33,34,35]. Elevated levels of inactive MGP, particularly the dephosphorylated-uncarboxylated form (dp-ucMGP), are strongly associated with increased vascular calcification and adverse cardiovascular outcomes, especially in populations with chronic kidney disease or diabetes [35,36,37]. Thus, the loss of functional MGP disrupts the balance between calcification promoters and inhibitors, leading to both endothelial dysfunction and progressive vascular calcification.

**Figure 1:**
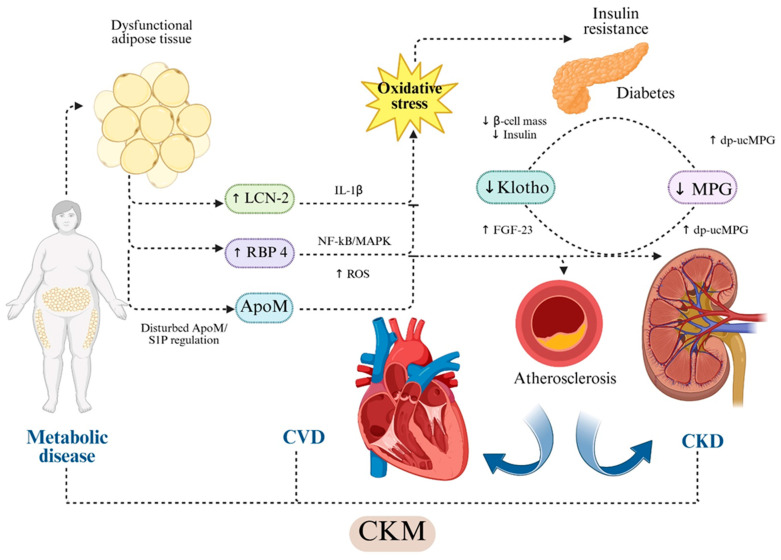
Hypothesis-based representation of cross-organ molecular pathways linking adipokine and vascular modulator dysregulation to CKM injury. Dysfunctional adipose tissue increases circulating RBP4 and LCN2/NGAL. RBP4 activates TLR4/NLRP3/NF-κB/MAPK signaling and induces oxidative stress, mitochondrial dysfunction and endothelial injury [25,41,42,43,44,45,46]. LCN2 promotes IL-1β-mediated inflammation, oxidative stress generation, NO reduction, MMP-9 activation and extracellular matrix remodeling [26,27,28,29,30,31,47,48,49]. ApoM, as HDL-bound carrier of S1P, maintains endothelial integrity and exerts anti-inflammatory and vasoprotective actions through S1PR1 signaling [32,33,50,51,52,53,54]. Soluble Klotho preserves endothelial NO synthesis, reduces ROS, modulates insulin signaling and regulates FGF23-dependent vascular effects [38,39,40,55,56,57,58]. Matrix Gla Protein (MGP) inhibits vascular calcification through BMP-2/4 and RUNX2 suppression; its inactive form (dp-ucMGP) reflects vitamin K-dependent loss of calcification control and is associated with vascular stiffness, CKD progression and adverse cardiovascular outcomes [33,34,35,36,37]. Arrows indicate published mechanistic directionality; dotted arrows represent indirect or context-dependent signaling. Dotted arrows indicate indirect or context-dependent signaling. The schematic highlights directionality and hierarchical relationships between molecular pathways. Abbreviations: ApoM—apolipoprotein M; CKD—chronic kidney disease; CKM—cardio-kidney-metabolic syndrome; dp-ucMGP—dephosphorylated uncarboxylated Matrix Gla Protein; eNOS—endothelial nitric oxide synthase; FGF23—fibroblast growth factor-23; IL-1β—interleukin-1β; LCN2—lipocalin-2/NGAL; MGP—Matrix Gla Protein; NF-κB—nuclear factor kappa-B; MAPK—mitogen-activated protein kinase; ROS—reactive oxygen species; RBP4—retinol-binding protein-4; S1P—sphingosine-1-phosphate; S1PR1—sphingosine-1-phosphate receptor-1. Created in BioRender. Gerdanovics, C. (2025). https://BioRender.com/kdqkfke.

In the presence of Klotho, FGF23 activates FGFR1, stimulating the Akt-eNOS pathway, which increases nitric oxide (NO) synthesis and promotes endothelial function (Figure 1). Klotho also enhances antioxidant defenses by upregulating superoxide dismutase 2 (SOD2) and catalase, counterbalancing FGF23-induced reactive oxygen species (ROS) production. When Klotho is deficient, FGF23-stimulated NO synthesis is blunted, and ROS degradation is impaired, resulting in increased oxidative stress and reduced NO bioavailability—key features of endothelial dysfunction [38,39,40].

Endothelial dysfunction in CKM syndrome is sustained by the combined effects of oxidative stress and inflammatory signaling. As presented, reduced ApoM-S1P and Klotho activity weakens antioxidant and vasoprotective defenses, while elevated RBP4 and LCN2 amplifyROS generation and impairs nitric oxide bioavailability. While MGP acts locally to inhibit calcium deposition, its inactive form removes this protective barrier, allowing calcification to proceed. Persistent oxidative stress and vascular inflammation, increases permeability, and triggers maladaptive remodeling, ultimately establishing a self-perpetuating cycle that accelerates cardiovascular injury.

#### Sex-Related Variability in Adipokine Signaling

Although adipokines share similar oxidative and inflammatory pathways across populations, several studies already included in this section suggest that circulating levels and biological effects may differ between sexes. RBP4 is closely linked to visceral adiposity and hepatic lipid metabolism, whereas LCN2 reflects inflammation-driven adipose remodeling. Since visceral adiposity predominates in males and inflammatory remodeling of subcutaneous adipose tissue is more common in females, these mechanisms may result in sex-dependent variability in circulating adipokine levels. Therefore, interpretation of adipokines in CKM should consider sex-specific adipose phenotypes rather than uniform thresholds across populations [28,29,30,31,32,33,34,35,36,37,38,39,40].

### 3.2. Lipid Dysregulation and Insulin Resistance in CKM Syndrome

Dysregulated lipid metabolism and insulin resistance are closely intertwined processes that play a pivotal role in the progression of CKM syndrome.

Beyond its classical retinol-transport role, RBP4 is a key signaling molecule linking adipose dysfunction to systemic metabolic impairment. Through activation of the TLR4–NLRP3 inflammatory pathway, RBP4 disrupts adipocyte insulin signaling and contributes to lipid and glucose dysregulation [41].

An additional mechanism by which RBP4 impairs insulin synthesis is acting directly at the transcriptional level through STRA6-mediated activation of the JAK2/STAT1 pathway in pancreatic β-cells. In pancreatic islets, STRA6 is specifically colocalized with insulin-producing β-cells, where it is upregulated by direct interaction with RBP4. This RBP4/STRA6 complex activates the JAK2/STAT1 signaling pathway, leading to STAT1-mediated repression of Isl-1 transcription, ultimately reducing insulin gene expression and impairing insulin synthesis [42].

oxLDL amplifies oxidative stress and activates redox-sensitive inflammatory pathways, including NF-κB and MAPK. This signaling environment alters adipokine secretion and reinforces adipose tissue inflammation. oxLDL also activates the NLRP3 inflammasome in macrophages, increasing IL-1β release, which disrupts insulin signaling in adipose tissue, muscle, and liver. Together, these processes create a feed-forward loop where dyslipidemia, innate immune activation, and adipokine imbalance perpetuate metabolic dysfunction in CKM syndrome [43,44,45,46].

A similar effect has been observed in patients with elevated Klotho protein level through NF-κB/ERK signaling, with Klotho overexpression in CKD-atherosclerosis models stabilizes plaques and lowers plasma cholesterol and triglycerides via macrophage ER stress pathways [55,56,57,58].

RBP4 is also associated with reduced clearance of VLDL-apoB100, positively correlating with triglycerides and large VLDL levels in patients with type 2 diabetes [43,59].

LCN2 activates the NLRP3 axis, sustaining adipose inflammation, impairing mitochondrial glucose handling, and contributing to insulin resistance. It also promotes foam-cell formation and modulates HDL flux via the Nedd4-1–SR-BI pathway, linking LCN2 to dyslipidemia and CAD [47,48,49]. Through the previously described NLRP3 inflammasome-dependent mechanism, LCN2 sustains adipose tissue inflammation and indirectly promotes insulin resistance by impairing mitochondrial function and glucose uptake in adipocytes. There is a positive correlation between NLRP3 and LCN2 expression, underscoring the pathophysiological relevance of this axis in metabolic dysfunction and insulin resistance [47]. LCN2 enhances macrophage scavenger receptor expression, promotes foam cell formation, and shows elevated circulating levels in individuals with greater CAD burden, linking it to dyslipidemia-driven atherogenesis. In the liver, LCN2 modulates HDL flux through the Nedd4-1-SR-BI axis, with experimental models showing that hepatocyte-derived LCN2 can reduce atherosclerosis, underscoring its direct influence on HDL metabolism and dyslipidemia [48,49].

ApoM plays a key regulatory role in insulin sensitivity through its modulation of S1P signaling pathways. S1P is a bioactive sphingolipid that interacts with five G-protein-coupled receptors (S1P1-S1P5), with S1P1 and S1P3 widely expressed in peripheral tissues and known to mediate protective effects against insulin resistance. Conversely, signaling through S1P2 has been associated with the promotion of insulin-resistant states. By facilitating the transport and bioavailability of S1P, ApoM indirectly influences the balance between protective and deleterious S1P receptor-mediated pathways, positioning it as a potential modulator of insulin sensitivity [50].

ApoM, carried on HDL, is a key determinant of HDL functionality, pre-β HDL formation, and cholesterol efflux. By chaperoning S1P, it modulates endothelial barrier integrity and inflammatory responses. In murine models, apoM overexpression enhances HDL function and cholesterol efflux, thereby reducing atherosclerosis. However, in the context of impaired LDL receptor pathways, it can elevate VLDL/LDL levels, highlighting context-dependent effects on lipoprotein metabolism. Conversely, apoM deficiency shifts hepatic and serum lipid profiles toward impaired lipid secretion, underscoring its mechanistic role in lipid handling [51,52,53,54].

Impaired Klotho function has been linked to aging-related metabolic dysfunctions, including insulin resistance. Mechanistically, Klotho appears to modulate insulin signaling by interfering with insulin-mediated phosphorylation events, reducing glucose uptake, lowering malonyl-CoA levels, and promoting fatty acid oxidation.

These effects contribute to decreased intracellular lipid accumulation, increased resistance to apoptosis and improved cellular longevity [60]. Klotho also exerts antioxidant and metabolic effects by modulating surface glycoproteins, including ion channels and receptors such as the insulin and IGF-1 receptors [61,62]. Soluble Klotho has been shown to inhibit the PI3K/AKT/mTORC1 pathway and upregulate peroxisome proliferator-activated receptor alpha (PPARα) via direct interaction with the IGF-1 receptor in high-fat diet-induced diabetic mouse models [63].

In CKM syndrome, these adipokines and vascular modulators interact through interconnected mechanisms of lipid dysregulation and impaired insulin signaling, accelerating atherosclerosis and metabolic deterioration. This creates a self-perpetuating cycle in which insulin resistance, dyslipidemia, oxidative stress, inflammation, adipokine imbalance, and calcific remodeling reinforce one another, ultimately driving cardiovascular events and CKM progression.

### 3.3. Tissue Remodeling and Vascular Calcification as Downstream Events

Tissue remodeling and vascular calcification represent downstream manifestations of prolonged inflammatory, oxidative and metabolic stressors in CKM. These adipokines and vascular modulators converge on common pathways that drive the terminal stage of vascular injury—remodeling and calcification. These molecules intersect at regulatory nodes controlling vascular smooth muscle cell (VSMC) phenotypic switching, mineral homeostasis and inflammatory signaling.

Klotho deficiency, prevalent in CKD and aging, increases vascular susceptibility to phosphate toxicity and promotes osteogenic reprogramming of VSMCs through BMP-SMAD-RUNX2 signaling, endothelial dysfunction and arterial stiffening [64,65,66,67,68]. Anti-fibrotic, anti-hypertrophic and anti-apoptotic properties have also been observed, exerting their effects through multiple signaling pathways involved in cardiac remodeling and heart failure. Among these, the interaction between fibroblast growth factor receptor 4 (FGFR4) and the calcineurin/nuclear factor of activated T-cells (NFAT) pathway is particularly relevant. In cardiomyocytes, FGF23 can activate FGFR4 in a Klotho-independent manner, initiating the calcineurin/NFAT signaling cascade. This activation leads to NFAT nuclear translocation and upregulation of pro-hypertrophic and pro-fibrotic genes, resulting in structural cardiac remodeling [69,70].

MGP is widely known for acting as a critical checkpoint in calcification control, with reduced activation linked to higher calcification burden [71,72,73]. In the setting of cardiac remodeling and heart failure—particularly when driven by hypertension—MGP plays a multifaceted pathophysiological role involving both vascular and myocardial compartments. MGP is a key regulator of extracellular matrix (ECM) homeostasis, and its deficiency or functional inactivity leads to pathological vascular remodeling. MGP expression is upregulated under increased wall stress and contributes to the preservation of microvascular and myocardial structure by inhibiting calcification and modulating ECM turnover. However, inactive form of MGP -dp-ucMGP -is associated with increased vascular and myocardial calcification, microvascular rarefaction and both perivascular and interstitial fibrosis. These alterations lead to increased myocardial stiffness and impaired diastolic relaxation—key contributors to the development of heart failure with preserved ejection fraction (HFpEF) [74,75]. Although MGP is not currently established as a clinical biomarker, its emerging role in cardiovascular remodeling suggests it may be considered in future diagnostic and prognostic frameworks.

LCN2 amplifies vascular injury through pro-inflammatory and oxidative stress pathways described before, fostering later VSMC osteogenic transformation and matrix vesicle-driven mineral nucleation [68,76]. LCN2 exerts pathological effects on cardiomyocytes, promoting hypertrophy via key signaling pathways while concurrently suppressing proliferation and reducing cell number. Both experimental models and clinical studies have demonstrated that circulating LCN2 levels correlate with increased cardiac mass, diastolic dysfunction and systemic inflammatory markers [76,77].

Elevated RBP4 can contribute to vascular calcification indirectly by promoting a pro-atherogenic, inflammatory and oxidative environment, as discussed before. These combined effects create conditions that drive vascular smooth muscle cells toward an osteogenic phenotype, supporting the biological plausibility of RBP4 as an upstream contributor to vascular calcification in CKM syndrome [78,79]. RBP4 contributes to cardiac remodeling and heart failure through its early described role in promoting inflammation and priming the NLRP3 inflammasome [41]. Also, RBP4 activates the TLR4/MyD88 signaling pathway in cardiomyocytes, leading to increased production of pro-inflammatory cytokines and reactive oxygen species (ROS) [80]. This inflammatory cascade induces the expression of hypertrophic genes, such as atrial natriuretic peptide (ANP), brain natriuretic peptide (BNP) and Myh7, ultimately promoting cardiomyocyte hypertrophy [43,81].

Collectively, reduced Klotho and active MGP, together with pro-inflammatory adipokines signaling—such as RBP4 and LCN2 -and impaired ApoM/S1P-mediated endothelial control, culminate in VSMC osteogenic transition, extracellular matrix mineral deposition and arterial stiffening—hallmarks of advanced vascular remodeling in CKM.

## 4. Metabolic Implications of CKM Molecular Pathways

Obesity represents a core component of the CKM syndrome, serving as a critical driver of the pathophysiological processes that underpin and exacerbate its associated comorbidities [1]. Dysfunctional adipose tissue is reflected systemically through altered adipokine profiles, among which RBP4 is a notable example. Circulating RBP4 levels are modulated by total fat mass and fat distribution, varying with body-weight changes over time [82]. Consistent with animal studies, RBP4 concentrations in humans are significantly associated with obesity parameters—including BMI, body fat mass, waist and hip circumferences, and waist-to-hip ratio (WHR)—in both pediatric [83] and adult populations [84,85]. Positive correlations have also been reported between RBP4 and visceral adipose tissue (VAT) volume, associated with increased cardiometabolic risk in obesity [82]. Importantly, VAT mass reduction—accompanied by improved insulin sensitivity—is associated with an approximately 25% decrease in circulating RBP4 in non-diabetic individuals with obesity [86]. Longitudinal data also show that circulating RBP4 closely tracks changes in fat mass during weight loss interventions but does not predict individual responsiveness [87]. In combined proteomic and metabolomic analyses of serum from children and adults undergoing bariatric surgery, RBP4 emerged as a significant predictor of body fat mass changes and overall adiposity [88,89]. Moreover, RBP4 also contributes to the pathogenesis of MAFLD by decreasing mitochondrial content and β-oxidation by inhibiting SIRT3, leading to increased LCAD acetylation and impaired fatty acid oxidation. This early mitochondrial dysfunction precedes the onset of steatosis and inflammation [82,90,91].

**Figure 2:**
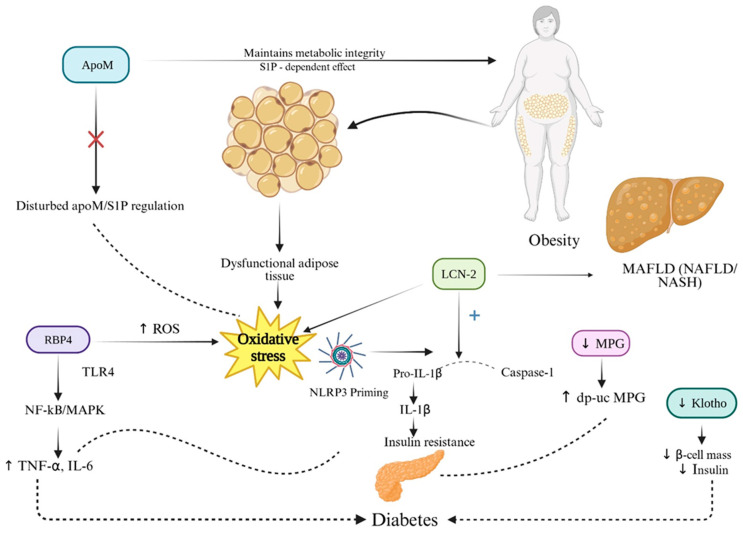
Adipokine and Vascular Modulator Interactions Driving Metabolic Dysfunction, Insulin Resistance, and MASLD in CKM Syndrome. In CKM syndrome, obesity-driven adipose tissue dysfunction alters adipokine secretion, promoting insulin resistance, dyslipidemia and hepatic lipid overload. Visceral fat-derived RBP4 impairs insulin signaling and enhances adipose inflammation via NF-Kb/MAPK. LCN2 also sustains adipose inflammation via NLRP3 priming and lead to MASLD. Reduced ApoM in obesity and metabolic syndrome impairs the ApoM-S1P axis, weakening metabolic protection and leading to insulin resistance. Low circulating Klotho is linked to insulin resistance and adverse metabolic profiles through FGF23-dependent pathways. MGP, as an adipocyte-secreted protein, associates with visceral adiposity and modulates adipogenesis, with its inactive form (dp-ucMGP) emerging as a biomarker of adipose dysfunction. Up and down arrows indicate published mechanistic directionality. Solid arrows indicate experimentally demonstrated pathway activation; dotted arrows indicate indirect or context-dependent signaling.Created in BioRender. Gerdanovics, C. (2025). https://BioRender.com/zg7hre3.

Genetic studies further support this association, with certain gain-of-function mutations in the RBP4 promoter linked to increased risk of obesity and type 2 diabetes (Figure 2). Elevated circulating RBP4 levels or increased RBP4 mRNA expression in adipose tissue are consistently found in obese and diabetic individuals, as well as in their first-degree relatives, correlating positively with both adiposity and insulin resistance [84,92].

Elevated LCN-2 expression is observed in the adipose tissue of obese individuals and is positively associated with obesity-related parameters (e.g., BMI, waist circumference, body fat percentage), and insulin resistance (higher fasting glucose levels, the HOMA-IR index) [93]. Circulating LCN-2 concentrations are significantly higher in obese compared to lean individuals, correlating with insulin resistance and inflammatory markers [94]. As a result, circulating LCN2 concentrations have been proposed as a potential inflammatory biomarker in obesity and related metabolic conditions [95].

The link between apoM and obesity has been extensively investigated and a recent study reported that APOM is expressed in human subcutaneous and visceral adipose tissue, predominantly by adipocytes. ApoM is released into the circulation from adipose tissue, and plasma apoM concentrations correlate with adipose tissue APOM mRNA levels. In adipose tissue, APOM expression inversely correlates with adipocyte size, being lower in obese individuals compared to lean counterparts and reduced in subjects with MetS. Also, it was observed that in obese individuals, caloric restriction increases APOM expression and secretion in adipose tissue [96].

In addition, several genetic variants of the APOM gene identified in the Chinese population have been associated with reduced plasma ApoM concentrations and an increased risk of developing type 2 diabetes [97]. Other studies demonstrated that circulating ApoM levels are significantly lower in individuals with obesity, metabolic syndrome, type 2 diabetes and gestational diabetes compared to lean, non-diabetic controls.

Recent studies have increasingly explored the potential role of the apoM-S1P axis in MASLD. Plasma proteomic analyses have confirmed significantly reduced apoM levels in MASLD patients, suggesting that disease-related dyslipidemia may be partly attributable to apoM deficiency [98]. In experimental studies it was demonstrated that apoM is an important regulator of hepatic lipid metabolism through modulation of autophagy [99].

The relationship between Klotho and conditions such as obesity remains incompletely understood. Studies suggest a negative association between circulating soluble Klotho levels and obesity, particularly among women. Notably, women who developed obesity early in life exhibited significantly lower soluble Klotho levels compared to their normal-weight counterparts. Thus, early-life obesity may induce long-lasting biological alterations affecting Klotho expression, potentially with sex-specific implications [100]. Similarly, research in school-aged children found that serum α-Klotho concentrations were negatively correlated with obesity-related parameters, especially in girls, further supporting this hypothesis [101]. Moreover, an inverse association between soluble Klotho levels and specific components of MetS, such as abdominal obesity has been observed, suggesting that soluble Klotho could serve not only as an early biomarker of metabolic risk but also as a potential mediator in the development and progression of metabolic disorders. Lifestyle factors, including regular aerobic exercise and adherence to healthy dietary patterns, appear to support the maintenance or even increase in soluble Klotho levels [102].

Experimental studies have shown that Klotho-deficient mice exhibit widespread tissue atrophy, particularly in the liver and adipose tissue [102] and reduced insulin secretion, despite the absence of obesity [103]. Interestingly, deletion of Klotho in leptin-deficient mice leads to reduced adiposity and enhanced insulin sensitivity [63]. Conversely, exogenous administration of α-Klotho in mice reduces fat mass, increases lean body mass, enhances energy expenditure and improves hepatic glucolipid homeostasis, even in the absence of changes in food intake or glycemia [104]. However, the precise balance by which Klotho modulates insulin sensitivity versus resistance remains incompletely understood.

Research on the relationship between Klotho and MASLD remains limited, though a positive correlation between Klotho and MASLD-related fibrosis may exist, due to the fact that a higher level of this protein has been observed in these patients. This fact may reflect a cytoprotective, compensatory upregulation aimed at mitigating cellular injury [105].

MGP is abundantly secreted by adipocytes, functioning as a novel adipokine, being upregulated during preadipocyte differentiation [106,107]. MGP regulates differentiation, lipid storage and lipolysis in 3T3-L1 cells, with serum dp-ucMGP levels positively correlating with visceral adiposity, suggesting that dp-ucMGP may possibly represent a novel serum biomarker for adipose tissue dysfunction and altered fat metabolism [108].

Additionally, MGP appears to rather influence the progression than the initiation of MASLD. Predominantly expressed in hepatic stellate cells, MGP modulates TGF-β signaling, a central driver of fibrogenesis. Hepatic MGP levels increase in parallel with fibrosis severity in both diet-induced MASLD mouse models and human liver samples. Notably, the antifibrotic effects of partial MGP deficiency occur without changes in hepatic lipid accumulation, supporting a role for MGP in fibrosis pathways rather than in the metabolic drivers of steatosis. Since liver fibrosis is a determinant of adverse outcomes in CKM, the involvement of MGP in this process underscores its importance in the progression of CKM-related comorbidities [109].

## 5. Molecular Pathways in Cardiovascular Dysfunction

CKM syndrome confers a significantly increased risk of cardiovascular complications, with studies suggesting that 45.3% of cardiovascular mortality risk associated with advanced CKM stages could potentially be prevented if individuals remained in non-advanced stages [110].

In patients with coronary artery disease (CAD), RBP4 levels are significantly higher than in those without CAD and positively correlate with the number of affected vessels. Some studies report reduced serum RBP4 levels in acute myocardial infarction and in men with familial hypercholesterolemia, where lower RBP4 predicted ischemic events within two years, suggesting a role in AMI [43,111]. Conversely, higher RBP4 in CAD patients has been linked to increased acute coronary syndrome events over a 3-year follow-up [112]. RBP4 has also been associated with essential hypertension, being correlated with left ventricular diastolic function [43]. Additionally, in elderly patients with chronic heart failure, circulating RBP4 levels were inversely correlated with left ventricular ejection fraction (LVEF) and positively associated with NT-proBNP concentrations. Moreover, serum RBP4 levels progressively increased with worsening cardiac function, suggesting its potential role as a biomarker of disease severity [113].

Elevated serum or urinary LCN2 levels are associated with atherosclerosis risk factors, disease severity and mortality, with studies suggesting that LCN2 could serve as a prognostic marker with patients presenting with acute coronary syndromes [114,115].

**Figure 3:**
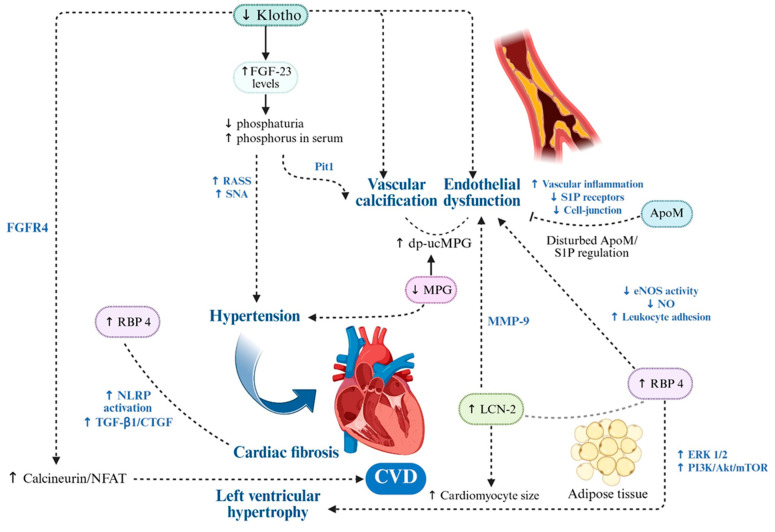
Molecular Drivers of Endothelial Dysfunction, Vascular Calcification and Cardiac Remodeling in CKM Syndrome. In CKM syndrome, cardiovascular injury arises from the interplay of pro-inflammatory adipokines and vascular modulators, which converge on pathways driving oxidative stress, endothelial dysfunction, vascular calcification and maladaptive cardiac remodeling. RBP4 and LCN2 amplify ROS production, impair nitric oxide bioavailability, activate NF-κB-dependent inflammation and promote matrix remodeling via MMP-9 activation, fostering both vascular stiffness and a pro-calcific environment. ApoM-S1P maintains endothelial barrier integrity and suppresses inflammation, but its reduction in CKM weakens vascular protection. Active MGP inhibits vascular calcification, while its inactive form (dp-ucMGP) accelerates osteogenic VSMC transformation, lead to hypertension and myocardial fibrosis. Klotho preserves endothelial function, antioxidant defenses and vascular elasticity, while its deficiency amplifies FGF23-driven vascular and myocardial remodeling. Collectively, these alterations initiate a feed-forward loop of vascular injury, calcification and cardiac structural changes that underpin the high cardiovascular risk in CKM. Up and down arrows indicate published mechanistic directionality. Solid arrows indicate experimentally demonstrated pathway activation; dotted arrows indicate indirect or context-dependent signaling. Created in BioRender. Gerdanovics, C. (2025). https://BioRender.com/g2h544e.

Clinical studies indicate that low serum Klotho levels are linked to increased arterial stiffness in patients with CKD and are independently associated with CAD severity in individuals with preserved kidney function. Furthermore, genetic studies suggest that Klotho gene polymorphisms may be associated with both longevity and CAD [116]. Klotho deficiency is mechanistically linked to the development of hypertension through several interconnected pathways involving FGF23, the renin–angiotensin–aldosterone system (RAAS) and sympathetic nervous activity (Figure 3). Excess FGF23, in the context of low Klotho, can exert off-target effects, including direct stimulation of sodium reabsorption in the distal tubule, promoting volume expansion and hypertension. Klotho deficiency upregulates multiple RAAS components, leading to increased angiotensin II and aldosterone production. Klotho directly suppresses aldosterone synthesis by downregulating adrenal CYP11B2 expression. Loss of Klotho thus results in increased aldosterone, sodium retention and hypertension [117,118]. Also, Klotho regulates central sympathetic outflow by modulating the activity of presympathetic neurons within the rostral ventrolateral medulla (RVLM). Through neuronal hyperpolarization, Klotho reduces sympathetic tone and contributes to blood pressure lowering [119].

Elevated levels of inactive MGP, particularly dp-ucMGP, have been associated with increased arterial stiffness and higher systolic blood pressure in population-based studies [120].

## 6. Molecular Drivers of the Renal Component

Renal involvement in the CKM represents a critical convergence of pathophysiological processes that interlink CKD, cardiovascular dysfunction and metabolic disturbances. The bidirectional interaction between the heart and kidneys is driven by a complex interplay of hemodynamic, neurohormonal, inflammatory and metabolic mechanisms, contributing to significant morbidity and mortality. As renal function progressively declines, the risk of adverse cardiovascular outcomes increases proportionally—and vice versa—while fibrosis and maladaptive tissue remodeling emerge as central contributors to disease progression [1,2,3]. Given the limitations of traditional biomarkers, there is increasing focus on novel molecular markers such as adipokines, Klotho, and MGP.

In recent years, adipokines have emerged as critical mediators linking adipose tissue dysfunction to renal pathophysiology, especially within the complex framework of the CKM syndrome, being now increasingly studied for their multifaceted effects on renal structure and function. Experimental and clinical studies have revealed their involvement in key pathophysiological mechanisms [121,122,123]. These findings underscore the pivotal contribution of adipokine imbalance—both excess and deficiency—in the initiation and progression of CKD, particularly when associated with obesity, insulin resistance and cardiovascular dysfunction.

The correlation between RBP4 and CKD is underpinned by the pivotal role of the kidneys in RBP4 metabolism. In circulation, RBP4 predominantly binds to transthyretin (TTR), a complex that prevents its glomerular filtration. Upon delivery of retinol to target tissues, RBP4 dissociates from TTR and becomes available for renal filtration, reabsorption and degradation within the proximal tubules. Notably, approximately 5% of circulating RBP4 remains unbound to TTR and is similarly processed by the renal tubules. Studies demonstrate that up to 99% of filtered RBP4 is reabsorbed in the proximal tubule, rendering urinary RBP4 a highly sensitive marker of early tubular dysfunction [124].

**Figure 4:**
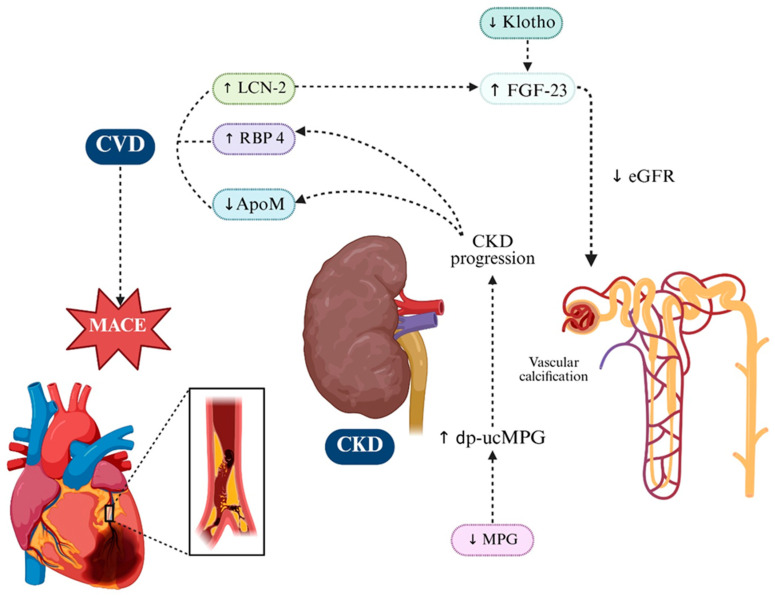
Key Molecular Players Driving Renal Injury and Progression in CKM Syndrome. In CKM syndrome, renal dysfunction arises from the convergence of pro-inflammatory adipokines and vascular modulators, which together accelerate CKD progression and its cardiovascular sequelae. RBP4 and LCN2 accumulate with declining eGFR. ApoM/S1P exerts renoprotective effects by maintaining endothelial integrity and limiting inflammation, while its depletion in CKD heightens susceptibility to injury. Klotho deficiency impairs phosphate handling, weakens antioxidant defenses, and—together with LCN2—drives FGF23 excess in a maladaptive loop linking renal and cardiac pathology. MGP, particularly its inactive dp-ucMGP form, reflects vitamin K-dependent calcification control loss, correlating with vascular stiffness and renal decline and poor outcomes. These interconnected mechanisms fuel progressive loss of kidney function in CKM. Up and down arrows indicate published mechanistic directionality. Dotted arrows indicate indirect or context-dependent signaling. Created in BioRender. Gerdanovics, C. (2025). https://BioRender.com/19p13um.

In the early stages of CKD, both impaired glomerular filtration and reduced tubular reabsorption contributed to elevated serum and urinary RBP4 levels. Consequently, renal function is considered a major determinant of circulating RBP4 concentrations, which tend to accumulate in the context of metabolic disorders such as diabetes and obesity. Clinical data support this relationship: diabetic patients with microalbuminuria exhibit significantly higher plasma RBP4 levels and inverse correlations have been observed between estimated glomerular filtration rate (eGFR) and serum RBP4. As eGFR declines, serum RBP4 progressively increases (Figure 4) [122]. In addition, increased RBP4 promotes renal and vascular inflammation via activation of pro-inflammatory pathways, endothelial dysfunction and oxidative stress described before, all of which contribute to the progression of CKD in the context of CKM syndrome. These findings underscore the strong interdependence between renal function and RBP4 homeostasis, suggesting a dual role for RBP4 as both a biomarker and a potential mediator of CKM-related renal dysfunction.

Another important adipokine implicated in CKM-related renal dysfunction is ApoM. Clinical studies have shown that ApoM levels are inversely correlated with CKD severity across mixed etiologies [125]. Although direct clinical data linking S1P to kidney function are limited, experimental studies have extensively investigated its role in renal pathology. S1P signaling has been shown to exert protective effects in models of both acute and chronic kidney injury, primarily by preserving endothelial integrity, reducing inflammation and limiting tubular apoptosis [123].

LCN2 plays a dual role in CKD as both a biomarker and a mediator of injury. As a biomarker, LCN2 is markedly upregulated in injured renal tubular epithelium and is readily detectable in urine and blood, where elevated levels correlate with reduced eGFR and faster CKD progression. Urinary LCN2 isoforms show strong associations with markers of kidney dysfunction, supporting their value for risk stratification beyond acute kidney injury settings [121,126,127,128]. As a mediator, experimental and translational studies demonstrate that LCN2 actively contributes to CKD progression. In subtotal nephrectomy and proteinuric models, genetic deletion of Lcn2 attenuates tubular apoptosis, interstitial damage, proliferation and mortality. Mechanistically, LCN2 acts downstream of EGFR-HIF-1α signaling to promote maladaptive tubular proliferation. LCN2 also impairs tubular cell bioenergetics via mTOR activation, mitochondrial loss and DRP1-mediated fragmentation—changes reversed by Lcn2 inactivation [128,129,130].

Recent findings have also revealed that circulating LCN2 levels are significantly elevated in both human patients and mouse models of CKD and correlate with excess FGF23 production [131]. Notably, the kidney-derived LCN2 appears to act as a signaling molecule that stimulates FGF23 synthesis in bone tissue (Figure 4). In murine models, administration of LCN2 increased bone Fgf23 mRNA expression and raised systemic FGF23 levels, supporting the existence of a kidney-to-bone axis whereby LCN2 may drive FGF23 excess in CKD [132].

The Klotho gene encodes a type I transmembrane protein that is primarily expressed in the kidneys. This protein features a large extracellular domain, a transmembrane segment, and a short intracellular portion, and it plays a crucial role in renal physiology and mineral metabolism [14]. In CKD, circulating levels of FGF23 and parathyroid hormone (PTH) progressively increase as renal function deteriorates, likely to preserve phosphate homeostasis via enhanced urinary phosphate excretion. Notably, FGF23 levels tend to rise earlier than PTH, suggesting a dominant role in phosphate regulation during early CKD progression [22].

Importantly, Klotho gene expression is significantly downregulated in the kidneys of CKD patients [133]. In early CKD stages (stage ≤ 2), serum and urinary Klotho levels begin to decline, followed by a compensatory increase in FGF23 concentrations [134]. The degree of urinary Klotho reduction has been positively correlated with the severity of estimated glomerular filtration rate (eGFR) decline. Elevated FGF23 levels have been independently linked to poor renal outcome [134,135]. These aspects could particularly be beneficial in identifying patients with early CKD at risk of disease progression.

Consequently, understanding the complex interplay between Klotho deficiency and FGF23 dysregulation is vital in the pathogenesis and progression of CKM. Serum Klotho has emerged as a potential biomarker for identifying high-risk individuals and predicting disease progression and mortality in CKM. Furthermore, both Klotho and LCN2 converge on the FGF23 axis, a critical pathway in the progression of CKD and its cardiovascular complications. This dual influence creates a feed-forward loop of mineral metabolism imbalance, vascular dysfunction and accelerated CKD progression, underscoring the therapeutic potential of targeting the Klotho-LCN2-FGF23 axis in CKM.

In the context of CKD, renal MGP expression appears to increase with disease progression. This upregulation correlates with worsening renal outcomes, rather than offering protection, suggesting a maladaptive or compensatory response to persistent tissue injury and mineral imbalance [136]. The accumulation of inactive MGP isoforms, particularly dp-ucMGP, reflects both systemic vitamin K deficiency and the severity of vascular and renal damage. This makes it a valuable biomarker for monitoring disease progression and cardiovascular risk in CKM syndrome [36,37,137].

Cross-sectional studies in CKD populations have demonstrated that estimated glomerular filtration rate (eGFR) is inversely correlated with plasma dp-ucMGP levels and positively with total ucMGP concentrations [138,139]. Moreover, in individuals with preserved renal function (eGFR ≥ 60 mL/min/1.73 m^2^), high baseline dp-ucMGP levels were predictive of incident decline in kidney function, reinforcing the hypothesis that active MGP may exert renoprotective effects [140].

## 7. Therapeutic Targeting of Pathophysiological Pathways in CKM Progression

Given the complex, overlapping mechanisms driving CKM-related metabolic, cardiovascular and renal dysfunction, non-traditional biomarkers such as RBP4, LCN2, ApoM, Klotho and MGP should be investigated not only as predictors of progression but also as therapeutic targets. Recently, RBP4 antagonists and inhibitors of its protein synthesis have been developed as potential strategies to modulate RBP4 activity and its downstream effects. RBP4 antagonists, such as BPN-14136, have demonstrated therapeutic potential in preclinical models of metabolic and cardiovascular disease, with more inhibitors currently being under evaluation (Table 1). Nevertheless, no RBP4-targeted therapies have currently been approved for metabolic, cardiovascular or renal indications. Although certain antidiabetic drugs appear to reduce RBP4 levels, conclusive clinical evidence supporting their efficacy for this specific purpose remains insufficient [141].

LCN2 has been investigated as a therapeutic target in cardiovascular and metabolic disease, but no LCN2-directed therapies have reached clinical approval. Preclinical studies suggest that modulating LCN2 may influence inflammation and cardiac remodeling, but translation to human therapeutics remains in early stages [76].

ApoM is currently under investigation for its anti-inflammatory and endothelial-protective properties. Preclinical studies using an ApoM-Fc fusion protein have demonstrated promising results, including attenuation of fibrosis and enhancement of endothelial function. However, no ApoM-targeted therapies have been approved to date for clinical use [142].

Therapeutic strategies aimed at enhancing Klotho expression—such as the use of renin-angiotensin system inhibitors, statins, vitamin D receptor agonists and experimental gene therapy are currently being explored in the context of chronic kidney disease and age-related disorders (Table 1). Several clinical trials are ongoing, while gene therapy approaches remain in the preclinical phase of development [143].

Although MGP has been widely studied in the context of vascular and renal disease, no therapies specifically targeting MGP have been approved to date. Ongoing research is focused on identifying pharmacological agents capable of modulating MGP activity to confer cardiovascular and renal protection, though such approaches remain investigational at this stage. While preliminary studies have identified strong associations between these markers and adverse clinical outcomes, interventional research remains limited.

Therapeutic strategies aiming to modulate their expression are being explored at experimental level, but clinical translation remains premature. Therefore, further mechanistic studies and translational trials are needed to validate whether they could represent actionable targets this high-risk population.

### 7.1. Assay Biology and Preanalytical Considerations

Interpretation of circulating and urinary levels of RBP4, LCN2/NGAL, ApoM, Klotho and MGP is strongly influenced by assay biology and preanalytical determinants. LCN2 exists as monomeric and heterodimeric isoforms, with urine and plasma reflecting different pathophysiological sources, while renal clearance and systemic inflammation markedly influence its circulating concentration [76]. RBP4 circulates partially bound to TTR, a complex that prevents glomerular filtration; only unbound RBP4 is filtered and reabsorbed, which affects plasma versus urine measurements [78]. ApoM is predominantly HDL-bound and functions as a chaperone for S1P, with receptor-specific effects (S1PR1 vs. S1PR3) and currently no validated clinical cut-offs [96]. Klotho exists in membrane-anchored and soluble isoforms, and ELISA assays show poor inter-assay agreement, complicating comparability between studies [102]. MGP undergoes vitamin K-dependent carboxylation; its inactive form (dp-ucMGP) reflects impaired activation and is assay-dependent, influenced by vitamin K status and warfarin exposure [108]. Collectively, these preanalytical and methodological factors limit harmonization of values across studies and represent a major barrier to clinical translation of these biomarkers.Human clinical and epidemiological evidence

Beyond mechanistic and animal model data, several large-scale human studies demonstrate clinical relevance for these biomarkers. NHANES-based analyses show that lower circulating Klotho is associated with liver fibrosis and metabolic dysfunction in the general population [105]. Soluble Klotho levels are also lower in individuals with obesity, particularly in women and in girls with early-onset weight gain, showing sex-specific patterns [100,101,102]. For ApoM, human adipose tissue studies reveal reduced ApoM expression in obesity and metabolic syndrome, genetic variants linked to type 2 diabetes, and decreased plasma ApoM in MASLD [96,97,98]. RBP4 and LCN2 correlate with insulin resistance and cardiovascular disease burden, and elevated circulating levels predict adverse outcomes and event severity in CAD populations [93,94,95,111,112,113,114,115]. Likewise, dp-ucMGP levels are associated with arterial stiffness and predict decline in kidney function in population-based cohorts [120,137,138,139,140]. Collectively, these clinical datasets complement mechanistic studies and reinforce the translational relevance of these biomarkers in CKM.

### 7.2. Therapeutic Translation and Clinical Implications

Beyond preclinical proof-of-concept, several therapeutic strategies modulating these pathways are already being explored in humans. For RBP4, antagonists and inhibitors of its protein synthesis (e.g., BPN-14136) are under evaluation as disease-modifying agents, while antidiabetic drugs show reduction in circulating RBP4 levels [141]. ApoM-targeted interventions (e.g., ApoM-Fc fusion protein) are being investigated for endothelial protection and fibrosis attenuation [142]. For Klotho, multiple clinical approaches—including renin-angiotensin system modulators, statins, vitamin-D receptor agonists and experimental gene-therapy—are under evaluation in ongoing clinical trials supported by recent translational studies and reviews on emerging Klotho-enhancing therapies and their feasibility in humans [143].

Major translational challenges include limited bioavailability of recombinant Klotho, lack of selective small-molecule Klotho agonists, and insufficient clinical validation of RBP4 antagonists for cardiometabolic indications [141,143]. Despite these barriers, the convergence of molecular evidence and early clinical work suggests that adipokines and vascular modulators may progress from biomarkers toward actionable therapeutic targets within CKM.

## 8. Final Considerations

Given their possible involvement in key molecular pathways—ranging from oxidative stress and low-grade inflammation to endothelial dysfunction, altered mineral metabolism and tissue remodeling—RBP4, LCN2, ApoM, Klotho and MGP represent more than isolated biomarkers. These molecules reflect the convergence of renal, metabolic and cardiovascular pathology characteristic of CKM syndrome. However, current evidence is largely derived from heterogeneous cohorts, isolated mechanistic studies or small-scale observational research. The clinical utility of these markers remains limited by the absence of standardized cut-off values, poor comparability across assays and a lack of longitudinal validation in high-risk populations. In addition, the current literature does not provide harmonized reference ranges, clinical-grade cut-offs, or population-specific calibration for these biomarkers. Assay heterogeneity (serum vs. plasma matrices, ELISA vs. mass-spectrometry platforms, different antibody specificities) produces values that are not interchangeable across studies, limiting comparability. Importantly, no studies have yet demonstrated incremental predictive value over established clinical tools such as eGFR, albuminuria, SCORE2/Framingham-type cardiovascular scores, or KDIGO risk staging using metrics such as AUC improvement, Net Reclassification Index (NRI), Integrated Discrimination Improvement (IDI), or decision-curve/net-benefit analyses. As a result, claims regarding clinical deployment remain hypothesis-generating rather than actionable, and their use for decision-making requires prospective validation in clearly defined intended-use populations. Moreover, although some of these targets have shown therapeutic modulation potential in preclinical models, translational gaps persist, and no disease-modifying interventions directly targeting them have yet reached clinical implementation.

Despite strong pathophysiological plausibility, most evidence linking RBP4, LCN2, ApoM, Klotho and MGP to CKM is observational or based on mechanistic studies. Data from Mendelian randomization, incremental predictive value analyses (AUC, NRI/IDI) and interventional studies remain limited. Therefore, these molecules should currently be regarded as investigational biomarkers and potential modulators, rather than established clinical tools. Summarized data is presented in Table 2.

Although most available studies report positive associations between RBP4, LCN2/NGAL, ApoM, Klotho and MGP and CKM-related outcomes, this should not be interpreted as evidence of causality. The majority of published data are observational or mechanistic, making these biomarkers susceptible to reverse causality and residual confounding. Recent Mendelian-randomization analyses examining circulating metabolites and protein markers showed that some of the associations identified in observational cohorts do not translate into causal relationships when assessed through genetic instruments [144,145]. Similarly, previous MR analyses demonstrated that well-established observational associations may disappear when causality is tested, reinforcing the risk of over-interpreting correlational data [2]. In addition, recent CKM framework papers emphasize that, despite pathophysiological plausibility, no emerging biomarker has yet shown incremental clinical utility over standard risk stratification (SCORE2/KDIGO) or validated cut-off values for clinical deployment [6,146]. Assay heterogeneity further limits translation: Klotho measurements show poor inter-assay agreement and non-linear relationships across CKD stages [147], ApoM/S1P signaling demonstrates context-dependent effects without validated clinical thresholds [147], and NGAL/LCN2 shows context-dependent effects and links mineralocorticoid signaling to vascular fibrosis, further reducing specificity in multimorbidity [27]. Collectively, these data indicate that despite consistent positive associations, current evidence is exploratory, non-causal and without incremental predictive value, and these molecules should be considered investigational biomarkers rather than clinically actionable tools.

The conceptual relevance of our findings is supported by the recent American Heart Association (AHA) Presidential Advisory (2023), which formally redefined cardiovascular, renal and metabolic diseases as components of a single integrated continuum, termed the Cardiovascular–Kidney–Metabolic (CKM) syndrome [148]. This framework emphasizes that metabolic dysfunction, chronic kidney disease and cardiovascular disease should no longer be regarded as isolated entities, but as biologically interconnected stages of the same disorder. In line with this paradigm, our synthesis highlights that the dysregulation of adipokines and vascular modulators (RBP4, LCN2, ApoM, Klotho and MGP) converges on shared molecular hubs—oxidative stress, endothelial dysfunction, lipid dysregulation and vascular calcification—reflecting the cross-organ mechanisms described by the AHA. By integrating these biomarkers within the CKM continuum, our work supports the shift from organ-centric evaluation toward a biology-driven stratification that may enable earlier recognition of high-risk trajectories.

## 9. Conclusions

The complexity of CKM syndrome calls for a multidimensional approach to disease characterization, beyond conventional clinical markers. The integration of molecular biomarkers into research frameworks may enable earlier biological detection of subclinical dysfunction and refined phenotyping of CKM trajectories. Their potential clinical contribution will, however, depend on assay standardization, establishment of validated thresholds, and demonstration of incremental value over existing risk-stratification tools. Incorporating non-traditional molecules such as adipokines and vascular modulators into clinical investigation provides a promising opportunity to improve early diagnosis, prognosis assessment and therapeutic guidance, as these markers reflect the convergence of metabolic, inflammatory, vascular and renal pathways and may signal dysfunction before irreversible injury occurs.

Importantly, this integrative view shifts the focus from organ-centered disease management toward the shared molecular hubs linking adipose, vascular, renal and cardiac injury, enabling earlier detection and supporting mechanism-based, stage-specific interventions. Future research should advance from isolated biomarker associations toward the development of multi-marker panels capable of capturing CKM staging and dynamic disease transitions across the continuum of metabolic, renal and cardiovascular risk. Validation of these panels should rely on multi-omics approaches—proteomics, transcriptomics and metabolomics—coupled with longitudinal clinical follow-up to determine their predictive and therapeutic utility in real-world settings.

By systematically exploring these molecules in a translational framework, and by integrating biomarker panels with precision-medicine methodologies, CKM may progress from descriptive diagnosis to biologically informed prognostication and personalized intervention.

## Figures and Tables

**Table 1:** Comparative overview of the five adipokines/vascular modulators in CKM syndrome, summarizing pathogenic vs. protective roles, key signaling pathways and translational potential. Up and down arrows indicate mechanistic stimulation or inhibition. Horizontal arrows indicate published mechanistic directionality.

Molecule	Dominant Role in CKM	Key Pathogenic/Protective Mechanisms	Main Signaling Pathways	Translational Potential (Biomarker/Therapeutic Target)
RBP4	Pathogenic	↑ inflammation, ↑ oxidative stress, ↓ insulin signaling, promotes cardiac remodeling	TLR4 → NLRP3 → NF-κB/MAPK; STRA6–JAK2/STAT1	Biomarker (insulin resistance, CAD, CKD progression); early-stage pharmacologic inhibition under development (RBP4 antagonists)
LCN2/NGAL	Pathogenic	↑ inflammation, ↑ ROS & MMP-9, foam-cell formation, VSMC osteogenic transition; cardiomyocyte apoptosis	NLRP3 inflammasome; EGFR–DRP1–mitochondrial fragmentation	Biomarker (renal injury, AKI/CKD, CAD severity); therapeutic modulation still preclinical
ApoM (ApoM–S1P axis)	Protective	↑ eNOS, ↑ endothelial integrity, ↓ inflammation, supports cholesterol efflux	S1PR1 > S1PR3; S1P signaling	Potential therapeutic target (ApoM-Fc fusion protein); strong biomarker candidate for MASLD/metabolic dysfunction
Klotho (soluble + membrane-bound)	Protective	↑ antioxidant defenses, ↑ NO synthesis, inhibits FGF23 adverse effects, ↓ fibrosis	FGFR1–Akt–eNOS; inhibition of IGF-1/PI3K/mTOR	Biomarker (CKD progression, CV mortality); gene therapy and Klotho-enhancing drugs in trials
MGP/dp-ucMGP	Protective when active/Pathogenic when inactive	Active MGP ↓ vascular calcification; inactive dp-ucMGP reflects loss of vascular protection	BMP-2/-4 → RUNX2 osteogenic pathway	Biomarker (vascular calcification, CKD, CV risk); no direct MGP-based therapies yet

**Table 2:** Evidence map of therapeutic strategies targeting adipokines and vascular calcification modulators in CKM syndrome, including indication, model, assay, effect and study limitations. Up and down arrows indicate molecular dynamics.

Molecule	Indication/Pathological Target	Model/Population & Assay Used	Effect (Directionality)	Study Design	Main Limitations
RBP4	Insulin resistance, endothelial dysfunction, inflammation	Humans (T2DM/CKD); rodents; endothelial cells • ELISA/WB/qPCR	↓ NF-κB/MAPK/TLR4-NLRP3; ↓ ROS; impaired NO	Preclinical + observational	Heterogeneity of assays; no therapeutic inhibitor
LCN2/NGAL	Tubular injury, inflammation, oxidative stress	Human CKD; mouse Lcn2-/-; cardiomyocytes • ELISA/IHC/proteomics	↓ ROS; ↓ IL-1β; ↓ MMP-9 activity; ↑ NO	Mechanistic	Dual biomarker/effector, context-dependent
ApoM/S1P axis	Endothelial barrier protection, anti-inflammatory	Endothelial cells; HDL-bound S1P models • ELISA/MS/S1PR1 signaling assays	↑ S1PR1; ↓ VCAM-1/E-selectin; ↑ cholesterol efflux	Preclinical	No clinical trial; S1P signaling dose-dependent
Klotho	CKD progression, oxidative stress, vascular stiffness	Human CKD; mouse CKD; endothelial cells • ELISA/qPCR	↑ eNOS; ↓ ROS; ↓ FGF-23 signaling	Observational + preclinical	Inter-assay variability; no therapeutic agent
MGP/dp-ucMGP	Vascular calcification suppression	Human CKD/diabetes; VSMCs • dp-ucMGP ELISA/BMP2-RUNX2 assays	Active MGP ↓ calcification; high dp-ucMGP predicts stiffening	Observational	No interventional evidence

## Data Availability

No new data were created or analyzed in this study. Data sharing is not applicable to this article.

## Abbreviations

The following abbreviations are used in this manuscript:

AKT	Protein Kinase B
ANP	Atrial Natriuretic Peptide
ApoM	Apolipoprotein M
BMP	Bone Morphogenetic Protein
BNP	Brain Natriuretic Peptide
CKD	Chronic Kidney Disease
CKM	Cardio-Kidney-Metabolic
COX	Cyclooxygenase
CVD	Cardiovascular Disease
CYP450	Cytochrome P450
dp-ucMGP	Dephosphorylated-Uncarboxylated Matrix Gla Protein
ECM	Extracellular Matrix
eGFR	Estimated Glomerular Filtration Rate
eNOS	Endothelial Nitric Oxide Synthase
FGF23	Fibroblast Growth Factor 23
FGFR	Fibroblast Growth Factor Receptor
HDL	High-Density Lipoprotein
HFpEF	Heart Failure with Preserved Ejection Fraction
JAK2	Janus Kinase 2
LCN2	Lipocalin-2
LDL	Low-Density Lipoprotein
MAFLD/MASLD	Metabolic (Dysfunction)-Associated Fatty Liver Disease
MAPK	Mitogen-Activated Protein Kinase
MetS	Metabolic Syndrome
MGP	Matrix Gla Protein
MMP-9	Matrix Metalloproteinase-9
NF-κB	Nuclear Factor Kappa B
NO	Nitric Oxide
NLRP3	NOD-Like Receptor Family, Pyrin Domain Containing 3 (inflammasome)
oxLDL	Oxidized Low-Density Lipoprotein
PI3K	Phosphoinositide 3-Kinase
PPARα	Peroxisome Proliferator-Activated Receptor Alpha
PPARγ	Peroxisome Proliferator-Activated Receptor Gamma
RAAS	Renin–Angiotensin–Aldosterone System
RBP4	Retinol-Binding Protein 4
ROS	Reactive Oxygen Species
RUNX2	Runt-Related Transcription Factor 2
RVLM	Rostral Ventrolateral Medulla
S1P	Sphingosine-1-Phosphate
STAT1	Signal Transducer and Activator of Transcription 1
STRA6	Stimulated by Retinoic Acid 6 (membrane receptor)
T2DM	Type 2 Diabetes Mellitus
TLR4	Toll-Like Receptor 4
VLDL	Very-Low-Density Lipoprotein
VSMC	Vascular Smooth Muscle Cell
